# Hypervariable Region 1 in Envelope Protein 2 of Hepatitis C Virus: A Linchpin in Neutralizing Antibody Evasion and Viral Entry

**DOI:** 10.3389/fimmu.2018.02146

**Published:** 2018-09-27

**Authors:** Jannick Prentoe, Jens Bukh

**Affiliations:** ^1^Copenhagen Hepatitis C Program (CO-HEP), Department of Infectious Diseases, Hvidovre Hospital, Copenhagen, Denmark; ^2^Department of Immunology and Microbiology, Faculty of Health and Medical Sciences, University of Copenhagen, Copenhagen, Denmark

**Keywords:** hepatitis C virus (HCV), hypervariable region 1 (HVR1), viral entry, vaccine design, neutralization

## Abstract

Chronic hepatitis C virus (HCV) infection is the cause of about 400,000 annual liver disease-related deaths. The global spread of this important human pathogen can potentially be prevented through the development of a vaccine, but this challenge has proven difficult, and much remains unknown about the multitude of mechanisms by which this heterogeneous RNA virus evades inactivation by neutralizing antibodies (NAbs). The N-terminal motif of envelope protein 2 (E2), termed hypervariable region 1 (HVR1), changes rapidly in immunoglobulin-competent patients due to antibody-driven antigenic drift. HVR1 contains NAb epitopes and is directly involved in protecting diverse antibody-specific epitopes on E1, E2, and E1/E2 through incompletely understood mechanisms. The ability of HVR1 to protect HCV from NAbs appears linked with modulation of HCV entry co-receptor interactions. Thus, removal of HVR1 increases interaction with CD81, while altering interaction with scavenger receptor class B, type I (SR-BI) in a complex fashion, and decreasing interaction with low-density lipoprotein receptor. Despite intensive efforts this modulation of receptor interactions by HVR1 remains incompletely understood. SR-BI has received the most attention and it appears that HVR1 is involved in a multimodal HCV/SR-BI interaction involving high-density-lipoprotein associated ApoCI, which may prime the virus for later entry events by exposing conserved NAb epitopes, like those in the CD81 binding site. To fully elucidate the multifunctional role of HVR1 in HCV entry and NAb evasion, improved E1/E2 models and comparative studies with other NAb evasion strategies are needed. Derived knowledge may be instrumental in the development of a prophylactic HCV vaccine.

## Introduction

It is estimated that at least 2 million people become infected with hepatitis C virus (HCV) every year (1). The majority of these individuals will develop chronic infections adding to the more than 71 million chronically infected people worldwide, who are consequently at increased risk of developing liver diseases, such as cirrhosis and hepatocellular carcinoma (1, 2). HCV-related mortality is estimated at 400,000 people every year, and although direct-acting antiviral therapies with cure-rates >95% are now available, treatment is often not accessible for multiple reasons, including frequent occult infection and high cost (3, 4). Thus, the development of a prophylactic vaccine is required to control HCV worldwide, but this challenge has proven difficult owing in part to the complex measures HCV employs to avoid the host immune responses (5).

HCV is an enveloped, positive-stranded RNA virus of the *Hepacivirus* genus in the *Flaviviridae* family (6, 7). The genome is ~9.6 Kilobases and encodes 10 functional viral proteins from a single polyprotein. Virus structural proteins form part of the virus particle with the Core protein assembling into the viral capsid that protects the HCV genome, and envelope proteins 1 and 2 (E1 and E2) imbedded in the viral envelope as the heterodimeric glycoprotein complex, E1/E2 (8, 9). *In vitro* systems for studying the role of E1/E2 in HCV entry and neutralization have been developed. Cell culture infectious HCV (HCVcc) can be produced in cell lines of hepatic origin and yields particles that share many similarities with *ex vivo* derived HCV (10–12). HCVcc recombinants encoding at least the structural proteins Core, E1 and E2 of a given HCV isolate, but depending on the unique replication capabilities of the JFH1 isolate (13), typically do not require cell culture adaptive envelope mutations (14–19), thus making these HCVcc recombinants particularly useful in studies of entry and neutralization. Such recombinants, including marker viruses, have been developed for major genotypes 1–7 (2, 20, 21).

Another model, used primarily for the study of HCV entry and neutralization, is HCV pseudo-particles (HCVpp), in which lentiviral or retroviral particles harbor authentic HCV envelope proteins (22–24). However, these particles are produced in non-hepatic 293T cells and therefore lack lipoprotein-association, potentially introducing additional bias in the *in vivo* relevance of obtained results. For example, many studies have shown that HCV particles associate with apolipoproteins, mainly ApoE, ApoCI, ApoAI, and debatably, ApoB (25–30). This is likely explained by the fact that HCV hijacks the very-low-density lipoprotein (VLDL) production machinery of the infected hepatocyte for virion production (30). In fact, HCV particles from patients and HCVcc systems display low density in gradients due to similarities with VLDL, whereas this is not the case with HCVpp (31–34). A study found that ApoE decreased accessibility of E2 neutralization epitopes (35). In addition, both ApoE and ApoCI appear to facilitate rapid virus entry, which promotes neutralizing antibody (NAb) resistance by decreasing time spent in the extracellular environment (36–38).

Initial attachment of HCV to the target hepatocyte has been shown to depend on virion-associated ApoE interacting with cell-surface expressed syndecan-1, syndecan-2 and T cell immunoglobulin and mucin domain-containing protein 1 (39–41). Following attachment, the HCV particle interacts with important entry co-receptors, such as scavenger receptor class B, type I (SR-BI), and CD81 (13, 14, 18, 23, 42–45). In addition, HCV relies on additional co-receptors, such as low-density lipoprotein receptor (LDLr) (46–48) and the late-stage entry receptors claudin-I and occludin (49, 50). Most recently, cellular factors that modulate HCV co-receptor localization and possibly prime the cell for infection have also been described (51–55). While it has been reported that LDLr may facilitate non-infectious uptake of HCV (48), it seems clear that the receptor must play an important role in infectious uptake, as recently confirmed for a number of HCV co-receptors, including LDLr (56). In addition, one study found redundancy in HCV entry dependency for SR-BI and LDLr, suggesting some overlap in function (57). As will be reviewed in the following sections evidence is mounting that the early entry co-receptors LDLr, and particularly SR-BI, are involved in HCV antibody evasion, possibly in an interplay with CD81 (45, 58–61).

**Figure 1:**
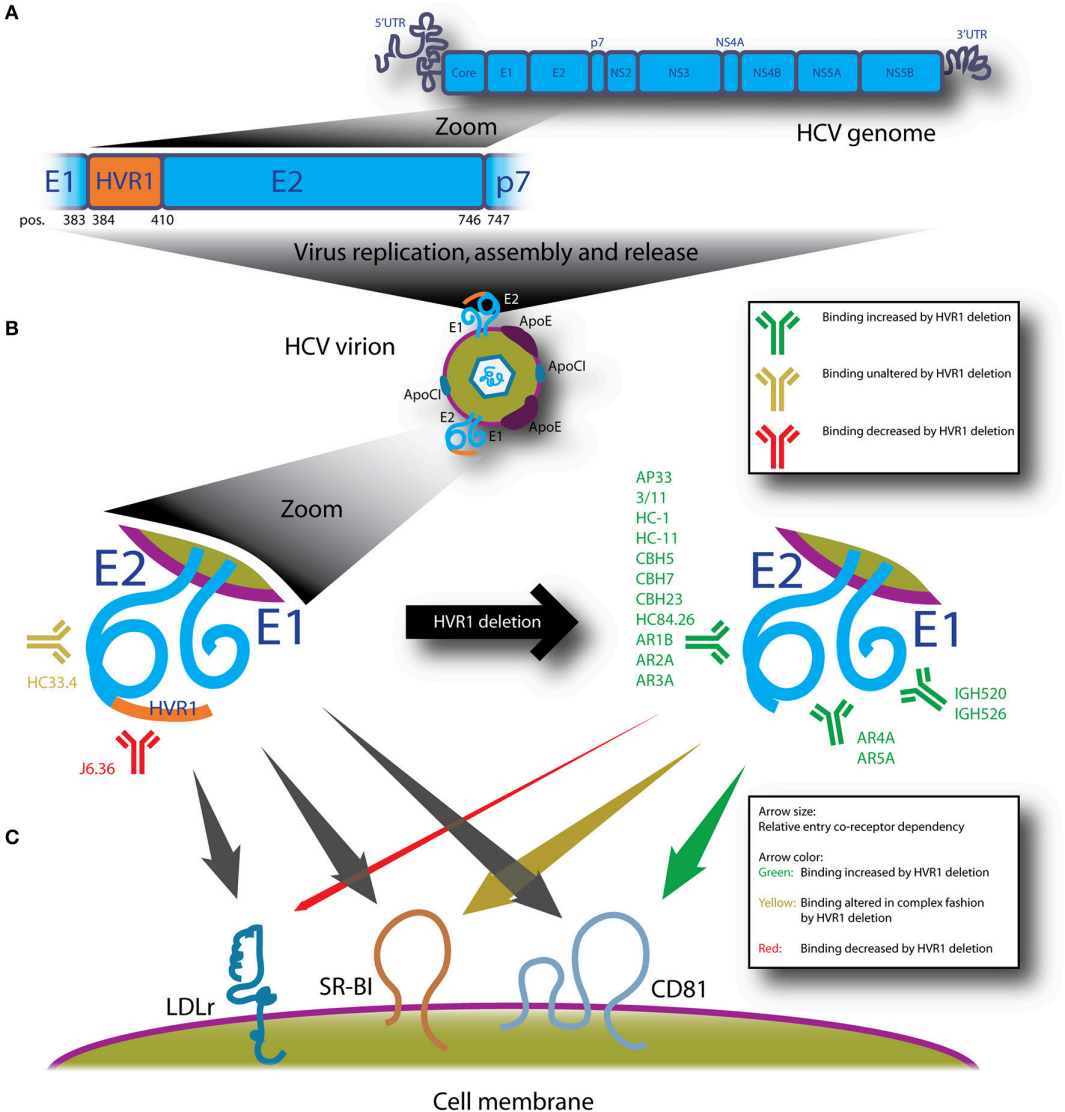
HVR1 of HCV is located at the N-terminus of E2 and protects the virus from diverse neutralizing antibodies and modulates entry interactions with LDLr, SR-BI and CD81. **(A)** Depicts HCV genome organization with a zoom of E2 showing that HVR1 corresponds to the 27 N-terminal amino acids of E2 (H77 reference sequence; amino acid position 384–410). **(B)** Replication of the HCV genome in a permissive cell leads to assembly and release of HCV virions with the E1/E2 complex embedded in the viral envelope. For HVR1-deleted HCV, sensitivity to NAbs is dramatically altered as compiled from multiple studies referenced in the text of this review. Monoclonal NAbs shown are part of comprehensive panels mentioned in the introduction and their specificities are: E1 (IGH520 and IGH526), E2; HVR1 (J6.36), E2; antigenic domain B (CBH5, HC-1 and HC-11), E2; antigenic domain C (CBH7 and CBH23), E2; antigenic domain D (HC84.26), E2; antigenic domain E/epitope I (AP33, 3/11, HC33.4), E2; antigenic region 1 (AR1B), E2; antigenic region 2 (AR2A), E2; antigenic region 3 (AR3A), E1/E2; antigenic region 4 (AR4A) and E1/E2; antigenic region 5 (AR5A). **(C)** HVR1-deleted HCV interacts differently with entry co-receptors LDLr, SR-BI and CD81, both in terms of dependency for entry (size of arrows) and how readily binding to the receptors occurs (color of arrow). Data is compiled from multiple studies cited in the text on the effects of deleting HVR1 from HCVcc, HCVpp or expressed forms of E2 or E1/E2.

Patient studies have found that an early induction of HCV-specific NAbs is correlated with resolving HCV infection (62–65). However, the virus employs mechanisms to avoid NAbs. The high mutation rate of HCV, due to the error-prone polymerase NS5B, permits continuous escape from NAb responses (66, 67). On a global scale, this heterogeneity has resulted in the emergence of six epidemiologically important genotypes and numerous clinically relevant subtypes (2, 6, 7). This has important implications for treatment and vaccine development, but this topic is outside the scope of this review. HCV also avoids NAbs by the capacity for cell-to-cell spread (68) and association with apolipoproteins as mentioned above (35–38, 69). Finally, HCV NAb sensitivity is intrinsically modulated by incompletely understood properties of E1/E2, such as envelope polymorphisms (70–73), N-linked glycans (the glycan shield) (74–77) and hypervariable region 1 (HVR1) at the N-terminus of E2 (58, 78, 79) (Figure 1A).

The study of the role of HVR1 in the HCV life cycle is a great example of how methodological breakthroughs advance and refine scientific questions. The development of HCVpp and HCVcc models (13, 14, 22–24), as well as the advent of novel tools, such as comprehensive panels of monoclonal antibodies with non-overlapping E1/E2 epitopes (80–91), have facilitated an increasing number of studies that improve understanding of the role of HVR1 in important aspects of the HCV life cycle, particularly immune evasion and viral entry.

## Characterization of HVR1 in patient studies

Shortly after the discovery of HCV, sequencing efforts identified the N-terminus of E2 as a hotspot of sequence variation, and it was termed HVR1 (92–94). The length of HVR1 was initially debated, but has since been agreed to be 27 amino acids long (amino acids 384–410 in the H77 reference strain), except for some subtypes of genotype 6 in which it appears to typically be 26 amino acids. In patients, HVR1 begins accumulating substitutions in the acute phase of infection (95, 96) and continues evolving during chronic infection (94, 97–99). The reason for this has been the subject of debate. One study, finding no evidence of positive selection and no correlation between evolutionary rate and HVR1-specific antibody responses in patients, suggested that random drift might be the cause for HVR1 variation (100). However, many studies did observe strong positive selection of HCV, particularly in HVR1 (101–103) and a large body of data now supports that HVR1 variation is due to antibody-driven immune selection. Firstly, antibodies against HVR1 are commonly detectable in chronically infected patients (37, 104–110) and the early induction of such antibodies is associated with acute self-limited infection (111). Interestingly, an early reduction in HVR1 sequence diversity is associated with acute self-limited infection (112), suggesting that a rapid anti-HVR1 response curtails virus proliferation before the virus is able to adequately establish a virus population in the host from which to adapt (e.g., diversify the HVR1 sequence). Secondly, although HVR1 variants are, at least to some extent, able to co-exist with the antibodies that recognize them (98, 106, 110, 113), emerging HVR1 variants have been found to have decreased reactivity with autologous patient serum antibodies, indicating that these variants represent escape (98, 106, 110, 113, 114). In addition, with the advent of the HCVpp entry model, HVR1 variants emerging in patients have been shown to be directly responsible for decreased *in vitro* neutralization with homologous serum (64). Finally, HVR1 variation is decreased or non-existent in HCV-infected patients with various types of immunoglobulin deficiencies (115–119).

The neutralization epitopes in HVR1, responsible for this antibody-driven hypervariability, seem to commonly reside in the C-terminus of the region (98, 106, 120, 121). Interestingly, despite the extremely high sequence diversity of HVR1, significant cross-reactivity of patient antibodies between HVR1 variants has been reported (104, 106–108). This may be because HVR1 contains highly conserved positions, such as conserved hydrophobic and positively charged residues, indicating functional constraints on HVR1 evolution (122).

## Characterization of HVR1 in studies of experimentally infected chimpanzees

Chimpanzees represent the first infection model of HCV and it has been used extensively to study HCV pathogenesis (123–125), including the role of HVR1. Incubation of hyper-immune serum raised against HVR1 peptide with a well-characterized homologous HCV chimpanzee inoculum prevented acute HCV infection in chimpanzees in one out of two cases (126), thus identifying HVR1 as the first HCV neutralization epitope. Interestingly, a minor variant of the inoculum had a different, serum-resistant, HVR1 sequence and this variant became dominant in the non-protected animal. It is therefore not surprising that anti-HVR1 antibodies in chimpanzees have been associated with HVR1 sequence variation (127), although HVR1 apparently does accumulate sequence changes more slowly in HCV-infected chimpanzees than it does in humans (128). This is likely due to subtle differences in HCV infection of chimpanzees compared with the human infection (129, 130), most notably the lower, and typically late, anti-HCV antibody response in chimpanzees (131).

Interestingly, it was possible to infect chimpanzees by intra-hepatic injection of HCV RNA with the HVR1 coding sequence deleted (132), resulting in acute infections, which in one case became an attenuated chronic infection. It was since shown that the animals had not raised NAbs and, in fact, that the chimpanzee that cleared acute infection with HVR1-deleted HCV could be chronically infected with the homologous virus following re-challenge (133). These studies confirm that NAbs are not critical for preventing chronic infection in chimpanzees and that HVR1 is not essential for HCV infectivity and persistence *in vivo*.

## HVR1 protects HCV from neutralizing antibodies

It was initially discovered that E2 expressed on the surface of cells did not appear to lose proper folding upon deletion of HVR1 (134). Subsequently it was shown that chimpanzees could be acutely and chronically infected with HCV by intrahepatic injection with HVR1-deleted HCV RNA transcripts, although infection was attenuated (132). With the advent of the HCVpp model of HCV entry it became possible to perform detailed studies of viral entry and neutralization (22, 23), but the deletion of HVR1 in the HCVpp model decreased infectivity 10 to 100-fold, making it challenging to study (44, 60). However, HVR1-deleted HCVpp was found to have increased susceptibility to NAbs targeting cross-subtype conserved epitopes (78), suggesting a role of HVR1 in NAb protection.

These studies were complemented with the advent of the HCVcc model (13, 14). The removal of HVR1 from HCVcc harboring E1/E2 from multiple isolates, including genotype 1–3, 5, and 6, had very different effects on culture viability (79). Some recombinant viruses were only slightly attenuated, whereas the fitness of others depended on one or two adaptive envelope substitutions, and the genotype 4a recombinant was non-viable (58, 79). Interestingly, while the H77(1a) envelope substitutions identified in the HCVcc model rescued infectivity of the HVR1-deleted H77 HCVpp, the opposite was true for HVR1-deleted S52(3a) HCVpp, in which the HCVcc adaptive envelope substitution decreased HCVpp infectivity even further (60). However, in all cases the resultant HVR1-deleted HCVcc displayed dramatically increased sensitivity to HCV NAbs and patient sera (58, 79). This phenomenon was initially believed to mainly involve epitopes that overlapped with the CD81 binding site of E2 (58), but it was recently shown that HVR1 protects a much wider variety of epitopes, such as antigenic regions 1–5 (AR1-5; on E2 and E1/E2), antigenic domains B-E (on E2) and even E1 epitopes (135) (Figure 1B). An exception to the broad increase in sensitivity is that viruses with and without HVR1 were similarly sensitive to the antigenic domain E antibody, HC33.4, and it has been suggested that this might indicate that HVR1 does not protect certain epitopes within antigenic domain E (136). However, it should be noted that HC33.4 has a secondary contact residue at position 408 within HVR1 (137), which could explain why HVR1-deleted viruses were not more sensitive to this antibody. The breadth in epitopes protected by HVR1 makes it less likely that direct steric epitope shielding alone accounts for the observed differences in NAb sensitivity of HCV with and without HVR1, but more studies are needed to address this in detail. Importantly, the ability of HVR1 to protect HCV from NAbs was recently confirmed *in vivo* by infusing HCV-permissive human liver chimeric mice with antibodies from a chronically infected patient prior to challenge using mouse pools of HCV with and without HVR1 (11).

The broad NAb-sensitizing effect of removing HVR1 has enabled the use of HVR1-deleted viruses to study virus escape in culture using lower doses of NAb than would otherwise have been needed (138). Although resistance substitutions identified in this manner for NAb AR5A were relevant for HCVcc retaining HVR1 (138), clear differences were observed in similar studies for NAb AR4A, which also appeared to have a higher barrier to resistance (139). It was recently found that HVR1-mediated NAb protection could be increased even further through the binding of HVR1-specific antibodies, possibly by increased steric occlusion mediated by HVR1-bound antibody (137). The HVR1-mediated NAb protection may function in concert with other highly variable region in E2 (140, 141), but how this interplay functions is largely unknown.

A related mechanism by which HVR1 has been proposed to protect HCV from NAbs is in serving as a decoy epitope, diverting the humoral immune system away from more conserved epitopes. This is supported by the correlation between persistence and higher non-synonymous to synonymous substitution rates in HVR1 (142), thus indicating that HVR1-directed immune responses can help the virus persist. The observed positive selection of HVR1 (101–103), combined with studies of HVR1 variants in immune-complexed HCV further supports this hypothesis (143, 144). In addition, the appearance of HVR1-specific antibodies in patient sera was associated with emergence of immune-complexes of particles carrying that specific HVR1 sequence, leading to a large reduction of that viral population within the patient (144). The idea that HVR1 contains immuno-dominant antibody epitopes with a high propensity for accumulating fitness-permissive escape substitutions fits well with the idea that HVR1 also protects other NAb epitopes on E1/E2. It could be hypothesized that immuno-dominance would be a possible consequence of the aforementioned epitope protection.

## *In vitro* studies of the role of HVR1 in HCV entry

SR-BI was identified as a possible HCV co-receptor by its ability to interact with soluble E2 (43). It was also found that HVR1-deleted soluble E2 protein lost most of the ability to interact with this receptor (43), although the interaction could be restored by the introduction of HVR1-deletion adaptive envelope substitutions previously identified *in vivo* (43, 132). These findings suggested that HVR1 modulates SR-BI interaction, but may not be directly interacting with SR-BI. The fact that an antibody against HVR1 blocked soluble E2 binding with SR-BI (43) is not proof of an HVR1/SR-BI interaction as the antibody could be sterically interfering with the SR-BI/E2 interaction without binding directly to the SR-BI binding site, much like the binding of antibody to an epitope tag on E2 neutralized tagged HCV (145). It was subsequently shown that HVR1-deleted soluble E2 more effectively bound CD81 (146). The advent of the HCVpp model confirmed CD81 (23, 44), and SR-BI (44) as co-receptors of HCV entry and facilitated in depth studies of their role in this process.

It was discovered that the human serum component, high density lipoprotein (HDL), enhanced HCVpp infectivity and this phenomenon was confirmed in multiple ways to be both HVR1 and SR-BI dependent (78, 147). In addition, HDL appeared to decrease NAb sensitivity of HCVpp (78, 148), possibly by increased speed of viral entry, thus minimizing the window during which neutralization could occur (149). These findings were corroborated in HCVcc studies (45, 148, 149). In parallel with these studies it was found that the HDL component, ApoCI, was sufficient to induce HCV infection enhancement (37). Interestingly, it appeared that ApoCI was transferred from HDL to HCV in an HVR1 and SR-BI dependent fashion, linked with the native lipid transfer function of the receptor (38). HDL does not interact directly with HCV in the absence of SR-BI (147, 149), but free ApoCI is able to do so, thus bypassing SR-BI (38). In fact, low doses of free ApoCI confer enhancement, while high ApoCI doses destabilize the virus, potentially through modulating virion fusogenicity (38).

SR-BI/HCV interaction was confirmed with HCV particles derived from human serum (150). However, this interaction did not depend on E2, but rather VLDL-like properties of these particles (150), most likely virion-associated ApoE. The fact that the interaction with SR-BI was energy-dependent and that suramin (a compound that reduces ApoE/receptor interaction) could not decrease the HCV/SR-BI association suggested that SR-BI might serve a role in endocytosis (150). However, the results could also be explained by secondary E2/SR-BI interactions, which might not be inhibited by suramin. HCVcc, which unlike HCVpp, is associated with apolipoproteins like ApoE, was used to address this possibility (151). It was found that the lipid-transfer function of SR-BI was critical for infection, but particles with densities above 1.1 g/ml depended on SR-BI specifically for cell attachment (151). While both these phenomena were independent of E2/SR-BI interaction, a third interaction involving a complex HVR1/E2/SR-BI/HDL interplay to enhance infectivity of HCV was also described (151), which is in line with findings from studies of HCVpp and HCVcc outlined above.

In addition, ApoE was found to be associated with HCV both with and without HVR1, but may serve different roles in the interaction with SR-BI (59, 60). While the nature of these differences remains unclear it is tempting to speculate that the high density HVR1-deleted particles interact with SR-BI through ApoE, as shown to be the case for high-density HCV retaining HVR1 (151). The fact that temporal blocking of CD81 and SR-BI yield similar HCV entry inhibition profiles may suggest that these HCV/receptor interactions are closely linked in time (45, 61), further stressing the possibility that SR-BI interactions lead to exposure of the CD81 binding site and downstream entry events.

It was found that the removal of HVR1 greatly increases accessibility of the CD81 binding site on E2 (58, 59). While HVR1 did not appear to modulate late-stage HCV entry co-receptor dependency for claudin-I and occludin, it did appear to influence the ability of HCV to interact with SR-BI (59). However, another study found that HVR1-deletion adaptive envelope mutations were responsible for altered SR-BI dependency as opposed to the deletion of HVR1 itself (60). Non-HVR1 E2 determinants of SR-BI binding would also be better in line with the fact that HVR1-deleted soluble E2 binding to SR-BI could be rescued by envelope mutations (43).

The part of HVR1 involved in modulating these processes, including the ability of HVR1 to protect HCV from NAbs, was since narrowed down to polyprotein positions 400–408 in the HCVpp model (121) and found to include conserved basic residues in HVR1, such as R408. However, in the HCVcc model it was also found that changing the N-terminal position 385 of HVR1 broadly influenced NAb sensitivity (152). It seems clear that intrinsic properties of the HVR1 sequence helps determine the level of HVR1-mediated NAb protection (120), but to what degree this depends on E1/E2 properties outside of HVR1 remains to be determined. Interestingly, many of the effects of removing or mutating HVR1 can be reproduced by the introduction of point mutations outside of HVR1 (153–155), suggesting the existence of non-HVR1 determinants.

HVR1 has also been proposed to interact with glycosaminoglycans in the HCVpp model, thus suggesting a role in attachment (156). However, the HCVpp model is typically deficient in ApoE, which is now believed to be the primary mediator of HCV attachment (39, 40), suggesting the results may not be as relevant for native HCV. Finally, HVR1-deleted HCV was shown to have decreased LDLr entry dependency (59, 60). In addition, HVR1-deleted HCVcc particles lost most of the ability to interact with soluble LDLr, suggesting a role of HVR1 in the interaction (60). Thus, HVR1 modulates the interaction of HCV with no less than three entry co-receptors (Figure 1C). Not surprisingly, several open questions remain, both with regards to receptor usage and NAb protection.

## Future perspectives for defining the role of HVR1

HVR1 apparently modulates interactions with no less than three HCV entry co-receptors, which may explain the functional constraints on HVR1 evolution. In addition, the inherent high variability of HVR1 permits it to serve as a rapidly changing decoy epitope, while directly protecting the virus from NAbs targeting a wide array of both conserved and less conserved E1/E2 epitopes. Not surprisingly, the deletion of HVR1 from soluble E2 protein fails to fully recapitulate these effects, which severely impairs reliability of molecular interaction studies and modeling. The structural flexibility of HVR1 has so far hindered crystallography studies of E2 protein retaining HVR1 (157, 158) and consequently we know very little about how this important region interacts with the remaining part of E2. Being able to produce and study a recombinantly expressed, native (i.e., as it sits in the virus membrane) E1/E2 heterodimer is urgently needed to further elucidate the contentious multi-functionality of HVR1 at a molecular level. The lack of native recombinant E1/E2 is also likely why the obvious interest in using HVR1-deleted vaccine candidates, in which conserved epitopes should be more exposed and consequently more immunogenic, has yielded conflicting results (159, 160). It is likely also evidence for the fact that HVR1 multi-functionality is dependent on the E1/E2 context on the virion. However, little is known about how much of the effect of HVR1 on NAb sensitivity and receptor dependency is intrinsic to the HVR1 sequence and how much depends on the E1/E2 context. In addition, the interplay between E1/E2 NAb protection caused by polymorphisms, N-linked glycans and HVR1 is virtually unknown. Such studies should offer a novel way to insights on how HVR1 serves its many functions, including the capacity to protect such a wide array of NAb epitopes.

The research on the role of HVR1 in the HCV viral lifecycle and host responses remains highly relevant, but despite great advances in our understanding of this unique genome region for HCV, particularly during the past 15 years, many questions remain. Providing answers to the role of HVR1 may prove critical in designing a successful HCV vaccine and stemming this global epidemic.

## Author contributions

All authors listed have made a substantial, direct and intellectual contribution to the work, and approved it for publication.

### Conflict of interest statement

The authors declare that the research was conducted in the absence of any commercial or financial relationships that could be construed as a potential conflict of interest. The reviewer FW and handling editor declared their shared affiliation at the time of review.
